# Diagnostic approach and arthroscopic treatment of septic arthritis of the knee, in an infant

**DOI:** 10.1002/ccr3.3382

**Published:** 2020-10-27

**Authors:** Nikolaos Laliotis, Chrysanthos Chrysanthou, Panagiotis Konstandinidis, Lambrini Giannakopoulou

**Affiliations:** ^1^ Orthopaedic Department Interbalkan Medical Center Thessaloniki Greece; ^2^ Radiology Department Interbalkan Medical Center Thessaloniki Greece

**Keywords:** arthroscopy, infant, knee septic arthritis

## Abstract

A 9‐month‐old baby presented with sudden inability to stand and unable to move his leg. Clinical examination showed edema and knee effusion. Blood tests and MRI confirmed septic knee arthritis without bone involvement. He was treated with arthroscopic lavage. He had a complete recovery and normal growth.

## Graphical abstract

Appropriate evaluation of clinical and laboratory findings combined with the MRI results in accurate diagnosis of septic arthritis in infants. Arthroscopic management is a convenient method, leaving a minimal scar.

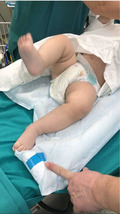

## 1 INTRODUCTION

Septic arthritis in an infant requires an accurate diagnosis and urgent surgical treatment. Prompt diagnosis is obscure in infancy. Babies are unable to point or localize their tendency. The main manifestations are the sudden onset of refusal to bear weight on the leg, the painful decreased mobility of the joint, the general irritability of the infant with concomitant signs with fever, sleep disturbance, and reduced appetite. In the clinical examination, there is joint effusion that is tender on palpation. It must be differentiated from other disorders like Lyme arthritis or trauma. If left with delayed diagnosis and treatment, severe deformities with a permanent lesion of the cartilage and deformities may follow. Diagnosis is supported with radiological examination with x‐rays and MRI investigation.^1^, ^2^, ^3^, ^4^

Appropriate treatment is urgent with surgical drainage of the joint, cultures for bacteria, and administration of antibiotics. Surgical treatment as an urgent procedure requires arthrotomy of the joint, thorough washing and in case of osteomyelitis drilling of the affected metaphysis. While arthroscopic washout is a regular procedure for adolescents and adults regarding the early management of septic arthritis, it is only recently becoming common in the pediatric population. The arthroscopic procedure was exceptional for infants, requiring small instruments and skills for the surgeon.^5^, ^6^, ^7^, ^8^

We present a 9‐month infant that was diagnosed with septic arthritis of the knee and was arthroscopically treated.

## 2 CLINICAL CASE PRESENTATION

**FIGURE 1:**
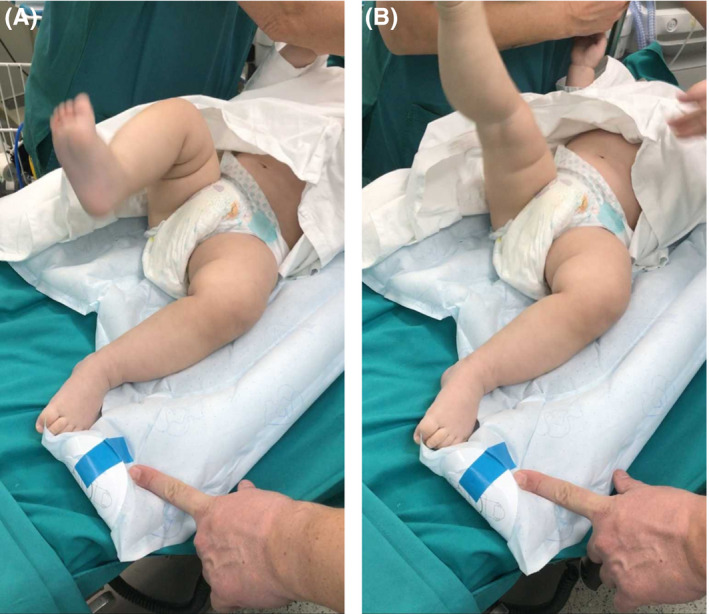
A, B Continuous kicking of the right leg and absent movements of the left leg

A 9‐month infant was referred to in our pediatric orthopedic department with acute symptoms during the last 2 days refusing to bear weight on the left leg. The infant had almost 3 weeks since he started to stand up. The parents informed us that the day before he had a fever of 38°C, but the day of the first examination the infant was afebrile. There was markedly reduced mobility of the left leg when in the supine position, with the continuous kicking off the right leg and reduced movements of the left leg Figure 1A,B (Video S1).

Clinical examination of the hip joint revealed a full range of movements, without any discomfort. Minimal edema of the medial knee joint area appeared, with discomfort when flexing and extending the knee. Immediate x‐ray was performed to rule out a fracture. X‐ray was normal without signs of periosteal elevation. Ultrasound of the hip was normal without elements of joint effusion. The ultrasound of the knee joint showed edema of the muscles, with joint effusion. Blood tests showed increased CRP and ESR. Neutrophils and platelets were increased. We performed blood cultures that were reported later negative Table 1.

**TABLE 1:** Blood tests measurements on admission

	ESR	CRP	WBC	Neutro	Lympho	Platelets
	40 mm	7.2 iu	15.4*1000 K/μL	4300/μL 28%	9800/μL 63%	855 K/μL
Normal	0‐10 mm	0.1‐1 iu	8‐12*1000 K/μL	1500‐4000/μL 20%	4000‐8000/μL 60%	200‐400 K/μL

**FIGURE 2:**
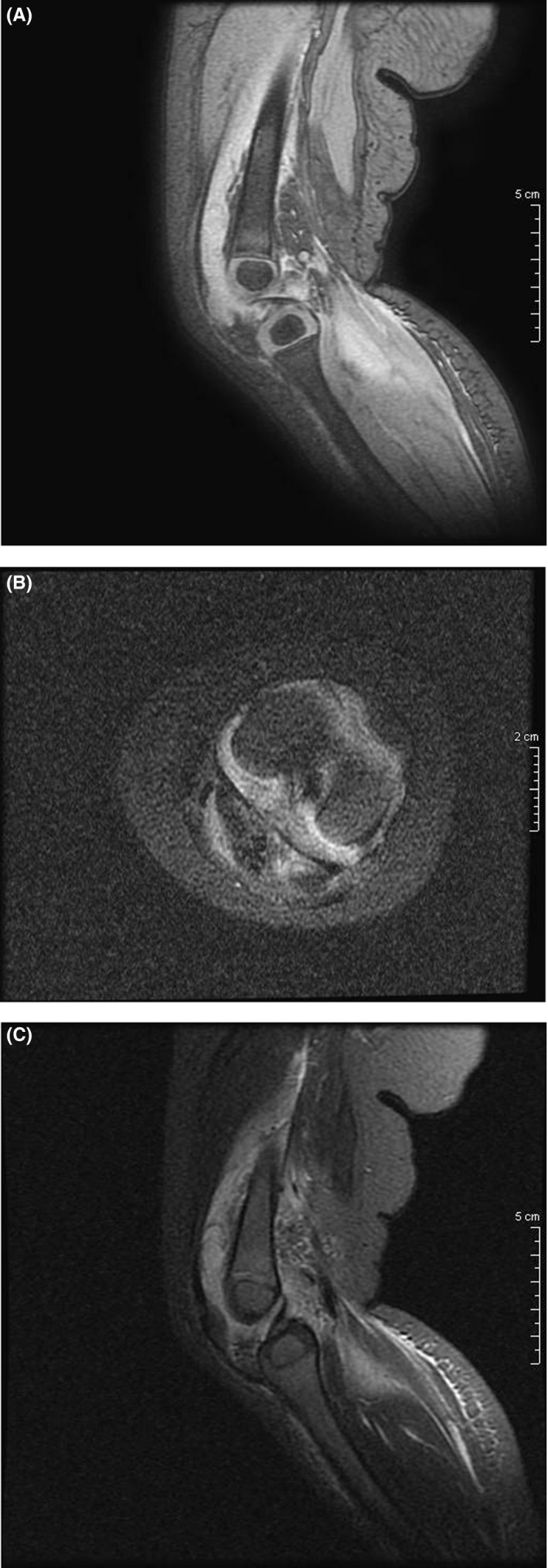
A, Sagittal STIR sequence demonstrates an extensive amount of fluid in the knee joint, a finding accompanied by edematous appearance of the muscles around the joint and a small amount of fluid in the intermuscular spaces. There is no apparent bone marrow edema. B, Axial STIR sequence. The intraarticular effusion is well delineated in the transverse plane, as well as the small amount of fluid around the muscles of the posterior compartment of the tibia. C, Sagittal MERGE sequence demonstrates intact articular cartilage of the knee joint

The infant had an MRI examination, under sedation. An extensive pathologic amount of fluid is observed in the left knee joint, with the involvement of the suprapatellar bursa. The finding is accompanied by the edematous appearance of the muscles around the joint (mainly the quadriceps and the medial head of the gastrocnemius), with a small amount of fluid in the intramuscular spaces. The lesions demonstrate intermediate to low signal intensity in T1W sequences and inhomogeneous high signal intensity in STIR sequences, without evidence of concomitant tumefactive lesion. There is no apparent edema of the bone marrow of the joint. The imaging findings in comparison with the clinical and laboratory findings mostly favor the diagnosis of septic arthritis, excluding evidence of osteomyelitis Figure 2A,B,C.

**FIGURE 3:**
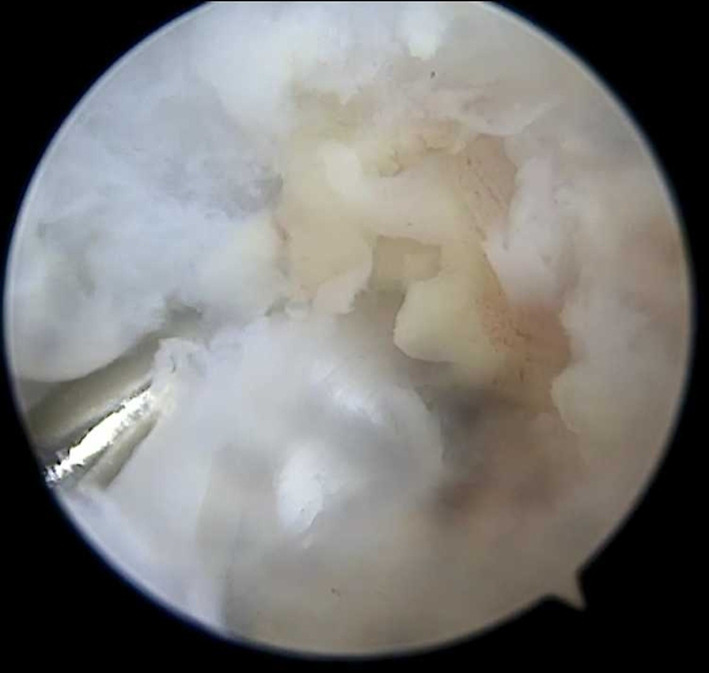
Arthroscopic picture with infected and hypertrophied synovium, in front of the ACL

Based on clinical, radiological, and laboratory examination, the infant was scheduled for knee arthroscopy. The infant was placed in a supine position. A standard anterolateral portal was established to introduce the 2.7 mm 30° arthroscope into the joint. An additional anteromedial working portal was performed. Introducing the scope in the joint, pus was pouring out from the cannula. That was sent for culture and microscopic analysis. The inflow of the pump was set to 30 mm Hg. A thorough lavage with 4.0 L of saline and inspection of the joint was followed. For the debridement of synovium remnants, a non‐traumatic 2.00 mm shaver was used. Joint examination revealed intact cartilage, meniscus, and cruciate ligaments, without signs of perforation from the metaphyseal area. Histological specimens from the synovial tissue were taken for examination. The procedure was completed in 20 minutes. We did not use drain because of the immediate and thorough lavage of the joint Figure 3.

The infant started immediately after the arthroscopy in intravenous antibiotics, with vancomycin. Cultures from the pus were negative for gram‐positive and ‐negative bacteria. Microscopic examination of the pus showed increased number of leukocytes (6000/μL, with 85% neutrophils). The pathological examination of the specimens confirmed the diagnosis of septic arthritis. There were specimens of the synovium with newly formed capillaries having hypertrophy of the endothelium. There were dense infiltrations exclusively with neutrophils.

A cast was used to immobilize the knee and relieve the infant for a week.

The infant had an immediate improvement in the clinical picture. In a week, he started standing on the affected leg even with the cast. There was an elimination of the edema, full range of knee movements in 2 weeks. Blood tests were performed with week intervals, with continuous improvement of CRP and ESR Table 2.

**TABLE 2:** Blood tests improvement, after treatment

	ESR	CRP	WBC	Neutro	Lympho	Platelets
In a week	33	1.9	24 K/mL	5000/mL 21%	17 300/mL 72%	606 K/mL
In 2 wk	22	1.5	18.7 K/mL	1900/mL 10%	15 500/mL 82%	425 K/mL

We stopped the iv antibiotics in 2 weeks and the infant started on oral antibiotics (amoxicillin clavulanate) for another 2 weeks. His blood tests were in normal values, and we stopped the use of antibiotics.

Six months later, the toddler is normally walking. He has normal blood tests. He remains in clinical evaluation in 3 months intervals. Scars for the portals are minimally visible on the knee.

## 3 DISCUSSION

Acute bacterial arthritis of the knee is a severe disease, and early diagnosis is essential to provide immediate treatment. Sequences from delayed diagnosis and inappropriate treatment have severe effects with cartilage and joint destruction and growth disturbances of the affected leg.

Septic arthritis in an infant always creates difficulties for the proper diagnosis. Inability to stand on the affected leg, discomfort, fever, irritability, and reduced mobility are the main clinical characteristics, but other diseases like an injury, a fracture, a Lyme monoarthritis, or a tumor may appear with the same clinical symptoms. Laboratory investigations including ESR, CRP, white‐blood‐cell count both peripheral, and synovial are helpful but none of them is diagnostic for septic arthritis.^1^, ^2^, ^9^

In children younger than 2 years with a history of fever, painful and reduced range of knee movements, and increased CRP >4 mg/L, diagnosis of septic knee arthritis is highly predictive.

Predictors for the septic hip arthritis do not accurately apply for the knee. Obey et al used Kocher's criteria in a cohort of 104 patients with a mean age of 8 years (0.1‐18.9 years). They report that 52% of patients could have missed the diagnosis. The sensitivity is increasing to 72% by adding an increase of CRP. They report that only 31.7% of patients had knee pain with reduced flection and extension of the knee.^3^ The accuracy of these diagnostic criteria is reported high in cases of culture‐positive septic arthritis but is lower in a negative culture.^10^

Arthrocentesis is used in knee effusion to perform cultures and examine the cells and IgG titers. Arthrocentesis of the knee joint is not easily accepted from young patients and in infants a type of sedation is commonly required. In a series of 189 patients younger than 18 years that arthrocentesis was initially performed, a high percentage of Lyme disease was diagnosed (140 patients). For the remaining 49 children that were diagnosed with septic arthritis of the knee, only 23 had positive bacterial cultures while the remaining were negative. All children with a diagnosis of septic arthritis had an urgent surgical incision for drainage of the knee joint. But in some children that were finally diagnosed as monoarthritis, the surgical procedure was unnecessary.^4^

It is important to establish an accurate diagnosis to avoid unnecessary operations. The diagnosis and treatment are challenging in infants because of nonspecific clinical symptoms and laboratory findings.

Negative bacteriological cultures in joint aspiration are not uncommon in infancy. In a series of infants <3 months, in 70% the cultures were negative. Saadi et al, report that 38% of patients had a true pathogen recovered in synovial fluid aspiration. In infants, staphylococcus remains the most common bacteria and thus despite that, the infant in our case had a negative culture, we administered vancomycin immediately after arthroscopy.^1^, ^2^, ^11^

Radiological examination is essential to exclude a fracture as a cause of the initial inability to stand with discomfort during movements. Ultrasonography is helpful revealing knee effusion but is not diagnostic. Magnetic resonance imaging is now easily accessible in all referral major hospitals. It requires sedation in infants but it has high accuracy. It can be combined with arthrocentesis at the time that the infant is in the examination area, sedated. Combined with the laboratory findings, it can provide high accuracy in diagnosis. Septic arthritis is characterized by joint effusion, with muscle involvement with edema. Most important is the exclusion of possible concomitant osteomyelitis in the metaphysis. In the presence of metaphyseal involvement, drilling of the bone is required. The periosteum may appear elevated in cases of infection or tumor. Tumor‐like involvement, like Ewing sarcoma or osteosarcoma or infantile types of leukemia, presents with chronic clinical signs and MRI findings show diffuse endosteal involvement with obscure limits, with extraperiosteal involvement. Rosenfeld et al suggest performing MRI, to exclude adjacent infections in cases of septic arthritis in children. Gibian et al analyzed 120 children over 6‐year period and underline the importance of immediate MRI imaging in increased CRP and older patients that are more likely to have osteomyelitis.^9^, ^12^, ^13^

In our infant, the early MRI investigation confirmed septic knee arthritis and the absence of osteomyelitis.

With the diagnosis of septic arthritis, urgent surgical drainage is essential. This was usually performed with an incision opening the synovium and lavage of the joint. It is impossible to inspect the whole knee joint with a parapatellar incision.

Arthroscopic lavage of septic arthritis of the knee in adolescents and adults is nowadays the method of choice. Since the development of small instruments, we can scope small joints of the hand and foot as well as peculiar joints like sternoclavicular or mandible joints.

Arthroscopy in infants requires appropriate small instruments but is superior to surgical incision since it permits easy inspection of the whole knee joint to exclude areas of cartilage destruction and possible osteomyelitis lesions that communicate with the knee joint. Introducing the scope in the joint, we performed examination and culture of the fluid. We performed biopsy of the infected synovium. Arthroscopy can be repeated easily in cases that infection insists or remains. Two minimal scars are left as signs of the procedure compared with the scar from arthrotomy that is commonly spreading in young children as it is perpendicular to the skin lines of the knee.

Arthroscopic treatment of septic arthritis in very young children is recently becoming popular. In a report of 24 children younger than 6 years, including nine knees with septic arthritis, the results were very encouraging without any complications from the procedure in the knee and no relapses. They were using a 3.5 mm arthroscope except for the ankles and a 3 weeks old baby for shoulder arthroscopy that they used a 2.7 mm arthroscope. Irrigation was performed with an adequate amount of saline.^5^ We used the smaller size 2.7 mm arthroscope since we were treating a 9 months old infant.

Comparing the arthroscopic lavage with an open arthrotomy, for septic hip arthritis, in older children, the results were similar, in a prospective randomized study.^14^

Arthroscopic treatment for septic arthritis for the knee had a success rate of 94% in a series of 56 children, with a minimum of 2‐year follow‐up. Six of them were younger than 1 year. Children had initially a knee aspiration under anesthesia, and when the macroscopic examination of the fluid was confirmed, they were immediately treated with arthroscopic lavage with a 5 mm arthroscope. Bacteriology was positive in 27 cases (48%). They used a drain for a mean time of 5 days (1‐9). They refer to two cases of in‐hospital recurrence that was similarly treated with a new arthroscopic lavage, and they have not performed any arthrotomies.^8^ In our patient, we performed immediate arthroscopy, and with adequate lavage of the joint, did not use a drain.

Johns et al report a study of 24 patients over a 20‐year period, with acute knee septic arthritis. 11 patients received arthroscopic irrigation and 13 an open procedure. One 6‐month‐old baby is the youngest that was treated with arthroscopy, while a 2‐month old is the youngest of an open one. They used a 2.7 mm arthroscope for the smallest children. Five patients required a second procedure in the open procedure group compared with none in the arthroscopic group. They report no differences in the long‐term follow‐up.^15^

The impact of a procedure with two minimal portals is better than an open surgical arthrotomy in an infant that suffers the minimal trauma possible.

## CONFLICT OF INTEREST

None of the authors has any conflict of interest.

## AUTHORS CONTRIBUTION

NL: is responsible for writing and editing the original draft. CC and NL: performed surgery and are responsible for the investigation and collection of data. KP: is responsible for the data collection and assisting in editing. LG: is responsible for radiological investigation.

## ETHICAL APPROVAL

Full consent has been given by the parents for publication of the case report.

## Supporting information

Video S1
